# A Dual-Modal Hybrid Marker for Automatic Registration and Coordinate Hand-Over in C-Arm CT-Guided Spinal Navigation

**DOI:** 10.3390/s26206433

**Published:** 2026-10-12

**Authors:** Taeho Kim, Sungmin Kim

**Affiliations:** 1 Department of Electrical, Electronic, and Computer Engineering, University of Ulsan, Ulsan 44610, Republic of Korea; hotakim@mail.ulsan.ac.kr; 2 School of Mechanical Engineering, University of Ulsan, Ulsan 44610, Republic of Korea

**Keywords:** hybrid marker, automatic registration, spinal navigation, C-arm CT, coordinate handover, target-proximal re-registration, target registration error

## Abstract

An asymmetric hybrid registration frame (RF) integrates radiopaque and optical markers to automate computed tomography (CT) registration and coordinate hand-over to a dynamic reference frame (DRF). We compared automatic and manual fiducial selection using three fixed CT datasets of a rigid sawbone phantom. Ten operators performed five manual registrations per CT, alongside one automatic benchmark per CT. Both methods used the same five registration fiducials, rigid registration procedure, and three evaluation centers excluded from transform estimation. Target registration error (TRE) was the root mean square distance between transformed design centers and CT-detected centers, without probe contact. Across equally weighted CT datasets, mean TRE was 0.538 mm for automatic and 1.565 mm for manual registration. The manual-minus-automatic difference was 1.027 mm (95% operator-bootstrap confidence interval, 0.951–1.148 mm). Mean registration processing times were 2.817 and 22.589 s, respectively. Under these common evaluation conditions, automatic fiducial detection reduced on-frame center discrepancy and registration time. These findings support the integrated registration and handover workflow as a basis for further validation at independent anatomical targets.

## 1. Introduction

Pedicle screw fixation is a standard technique for correcting spinal deformities and structural instability. The narrow pedicle lies close to critical structures, including the spinal cord and aorta, so screw insertion requires precise trajectories within extremely tight anatomical tolerances. Historically, conventional pedicle screw placement showed substantial rates of malposition, highlighting anatomical and technical constraints [1]. C-arm fluoroscopy remained limited by its two-dimensional imaging and by substantial radiation exposure to both the surgical team and the patient [2]. Multiple systematic reviews and meta-analyses have confirmed that computer-assisted navigation substantially improves screw placement accuracy over conventional techniques [3,4].

Image-guided surgery (IGS) systems combine three-dimensional (3D) imaging with instrument tracking to support navigation [5,6]. In a large-scale meta-analysis, Naik et al. reported a pooled pedicle screw placement accuracy of 96.2% for 3D fluoroscopic navigation, with misplacement rates comparable to robotic surgery [7]. Although robotic-assisted surgery has also emerged [8,9], recent evidence indicates that robotic systems are costly and have not demonstrated clear clinical superiority over conventional navigation in terms of complications or reoperation rates [10,11], motivating further efforts to optimize existing navigation workflows.

Current spinal navigation systems based on optical tracking systems (OTS) still face persistent limitations in both registration and tracking. In particular, the fixation approach used for the registration frame—whether invasive or non-invasive—and its spatial relationship with the surgical field are critical determinants of registration accuracy and intraoperative workflow efficiency.

First, the fixation method of the registration frame involves an inherent trade-off between accuracy and patient burden. Bone-implanted fiducial markers and reference frames clamped rigidly to the vertebra provide robust coupling to the skeletal anatomy. Implantation of bone fiducials can require skin incisions [12], which adds invasiveness and procedural burden [13]. In multi-level procedures, repositioning increases this burden when additional incisions are required. Non-invasive fixation with skin-mounted markers and operating table-based attachments reduces invasiveness but remains vulnerable to soft tissue deformation, respiratory motion, and vertebral displacement during screw insertion. Such motion and deformation can impair navigation accuracy during the procedure. Therefore, temporary placement of the registration frame (RF) must be distinguished from the rigid anatomical coupling required for the tracking reference used after registration.

Second, registration is a key factor in navigation accuracy independently of the fixation approach. A recent meta-analysis of optical neuronavigation across cranial and spinal procedures reported a reference target registration error (TRE) of 2.34 mm [14], underscoring the need to evaluate navigation accuracy beyond tracking hardware precision alone. Various registration approaches have been investigated to address this limitation, each with distinct trade-offs. Surface-based registration is widely adopted for its non-invasive workflow, but is prone to substantial positioning-dependent errors. In a cranial navigation setting, Furuse et al. reported significantly lower accuracy for surface-matching registration than for automatic registration using intraoperative 3D imaging. The difference was particularly evident in the prone and lateral positions, where skin deformation distorts the body surface contour [15]. Frisk et al. compared automatic image registration using intraoperative cone beam computed tomography (CBCT) with surface-matching registration in 39 spinal surgery patients. Automatic registration achieved significantly higher accuracy (1.20 ± 0.42 mm vs. 1.94 ± 0.64 mm, p<0.0001), whereas surface matching was susceptible to operator errors such as identification of the incorrect vertebral level [16]. Taken together, these findings support the accuracy advantages of automatic image registration over surface matching in the settings studied. Manual registration tasks such as fiducial localization [17] and virtual model alignment in augmented reality navigation [18] can introduce localization or alignment errors and increase procedural time. In fiducial-based workflows, automating fiducial detection and matching eliminates manual fiducial selection variability.

Third, automating detection addresses operator-dependent variability but does not resolve a separate and more fundamental geometric constraint inherent to fiducial-based registration. Registration accuracy is known to degrade as the distance increases between the fiducial configuration and the surgical target [19]. Placing the registration frame close to the target vertebra can reduce distance-related TRE, but a bulky frame fixed near the target segment can physically interfere with instrument trajectories and collide with the surgeon’s hands. Conversely, placing the frame farther away preserves the workspace but can increase TRE. In long-segment procedures, such as multi-level fusion for degenerative disease or deformity correction spanning ten or more vertebral levels, this distance dependency motivates local re-registration near successive targets; indeed, in a clinical series of 826 spinal and iliac screws, Scheufler et al. observed a trend toward reduced navigation accuracy with increasing distance from a single reference arc and advised a registration update using a more proximal reference arc position for instrumentation spanning more than 11 vertebrae [20]. Frisk et al. also described separate CBCT acquisitions at different vertebral levels in patients with long constructs [16], illustrating the use of level-specific imaging and registration. Re-registration becomes a substantial obstacle to surgical flow when it is slow, technically demanding, or requires additional surgical incisions. These limitations motivate rapid automatic re-registration using an RF placed temporarily near the surgical target, followed by coordinate handover to a dynamic reference frame (DRF) rigidly coupled to the target anatomy.

This study presents an asymmetric dual-modal RF, an automatic registration and coordinate handover workflow using its design geometry, and a comparison of automatic and manual fiducial selection under common conditions. The contributions are as follows:

- **Dual-modal RF implementation:** Four infrared reflective spheres and eight radiopaque CT markers are integrated into an asymmetric frame. Design center coordinates expressed in the registered RF coordinate system link the CT marker geometry to optical tracking.
- **Integration of automatic processing:** Candidate selection and correspondence based on the design geometry, rigid registration, and coordinate handover to a separate DRF are implemented within one workflow. The RF serves image registration, while the DRF provides the tracking reference after hand-over. Automatic CT marker detection removes the manual fiducial selection step and returns the same fiducial coordinates for a fixed CT dataset and detection settings.
- **Comparison under common conditions:** Automatic center detection and manual fiducial selection are compared using the same CT volume, five registration fiducials, rigid registration procedure, and three evaluation centers excluded from transform estimation. This design evaluates differences in on-frame center discrepancy and processing time associated with the method of determining fiducial coordinates in the fixed CT datasets.

In the proposed clinical workflow, the RF is placed near the surgical target on a supporting surface, such as skin or exposed vertebral bone, without adhesive fixation, clamps, or screws. The RF is positioned so that both it and the DRF can be tracked simultaneously when their relative pose is acquired for registration. It is lifted away after coordinate handover to the DRF, and may be repositioned near a subsequent target before a new CT acquisition and registration. The experimental evaluation maintained the phantom–RF–DRF configuration used for CT acquisition.

**Figure 1:**
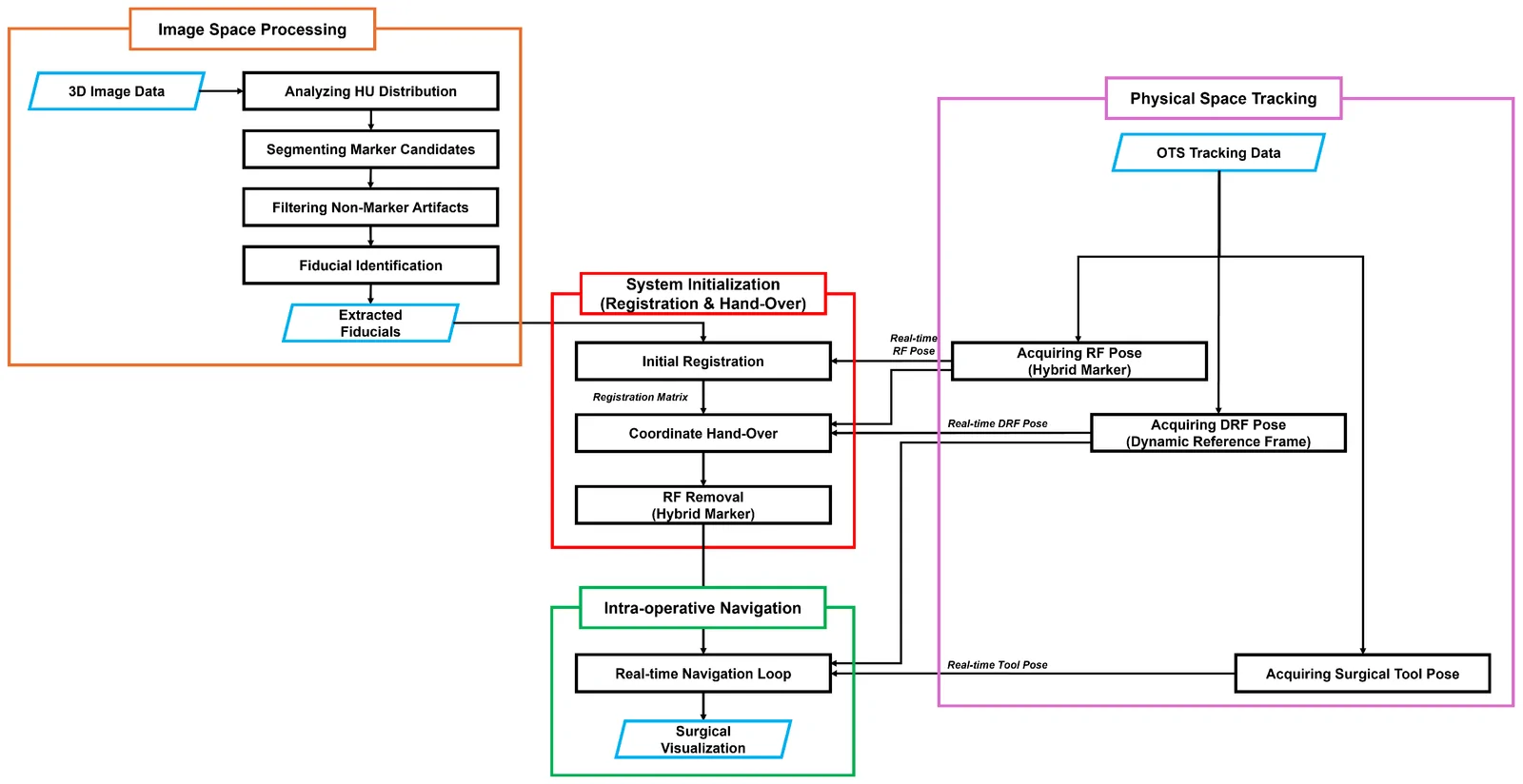
Proposed navigation workflow for the hybrid marker system. The system consists of four stages: image-space processing (orange), physical-space tracking via the optical tracking system (OTS) (pink), system initialization with registration and coordinate handover (red), and intraoperative navigation (green).

Figure 1 summarizes the proposed workflow. We describe its implementation and compare the fiducial registration error (FRE), on-frame center-based TRE, and processing time across three CT datasets. The common center-based evaluation compares design and CT-detected target centers using the recorded registration transforms, allowing the two fiducial coordinate determination methods to be evaluated without probe contact.

## 2. Materials and Methods

The proposed clinical workflow is intended to clear the surgical workspace by removing the hybrid marker after registration and coordinate handover to the DRF, while navigation continues using the DRF. The workflow has four stages, as shown in Figure 1. Image-space processing (orange) automatically detects and identifies CT markers in the 3D CT volume through Hounsfield unit (HU) distribution analysis, candidate segmentation, artifact filtering, and fiducial identification. Physical-space tracking (pink) runs in parallel: the OTS continuously tracks the poses of the optical marker frames and surgical tools in real time, supplying the tracking data needed by subsequent stages. During system initialization (red), the image-space fiducials and tracked RF-to-DRF relation are used to compute the DRF-to-CT registration. This stored registration supports subsequent navigation without a new RF input. Intraoperative navigation (green) uses the real-time DRF and surgical tool poses from the OTS to drive the navigation loop and display the tracked instrument in CT space.

### 2.1. Design of Hybrid Marker

**Figure 2:**
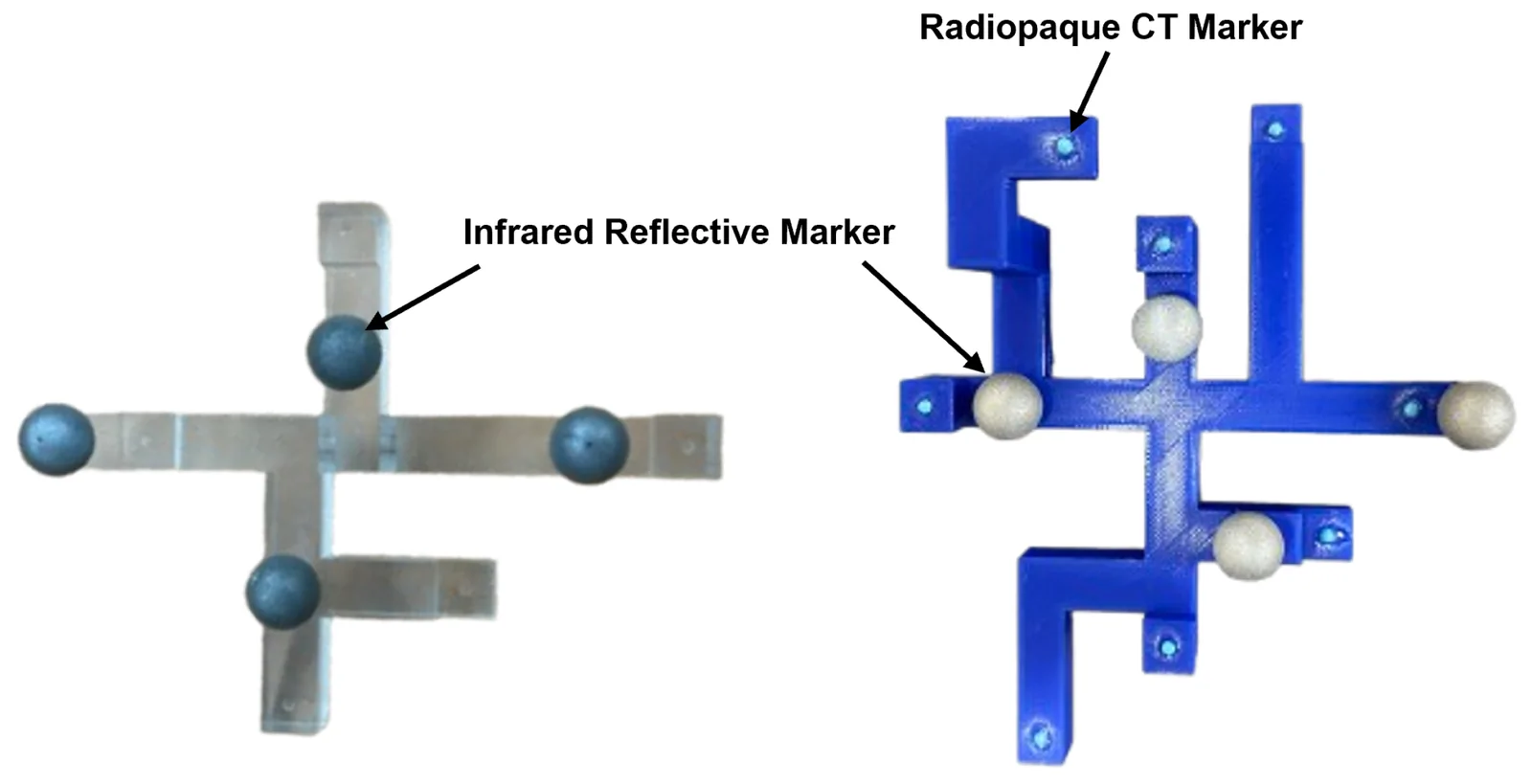
On the left, the dynamic reference frame (DRF) carries four infrared reflective markers for optical tracking. On the right, the hybrid marker (registration frame, RF) combines four infrared reflective markers with eight radiopaque computed tomography (CT) markers, supporting both tracking and image-based registration.

As shown in Figure 2, the system consists of two frames: the DRF (left), and the hybrid marker or RF (right). The DRF is a cross-shaped frame carrying four infrared reflective markers for real-time tracking by the OTS. The RF is an asymmetric frame that integrates four infrared reflective markers and eight radiopaque CT markers (Suremark CT 2.3, Suremark Co., Simi Valley, CA, USA, diameter 2.3 mm) into a single structure, thereby supporting both optical tracking and CT-image-based registration. The asymmetric geometry facilitates marker identification and pose determination. The nominal center coordinates of the eight CT markers are defined by the design geometry in the RF coordinate system. Their positions in physical space are computed from these coordinates and the RF pose measured by the OTS.

For optical marker registration, the four infrared reflective spheres on each fabricated frame were observed and registered with the OTS. Using the sphere configuration as the reference, the frame origin was adjusted to the intersection of the frame members and its axes were aligned with the corresponding design coordinate system. The CT-marker design coordinates are expressed in this RF frame. The same marker definition files were used on the experimental computer.

After fabrication, a Vernier caliper was used to compare frame dimensions, thicknesses, and center-to-center marker distances with their design values.

### 2.2. Navigation Workflow Using Registration and Tracking Frames

The proposed registration and tracking frame workflow involves four principal coordinate systems: the CT image coordinate system {CT}, the OTS coordinate system {OTS}, the hybrid marker coordinate system {RF}, and the dynamic reference frame coordinate system {DRF}. The transformation $T_{B}^{A}$ denotes a 4×4 homogeneous transformation matrix that maps coordinates from frame {A} to frame {B}.

The coordinate construction comprises image registration, coordinate handover, and instrument projection. The implementation obtains the RF-to-DRF relation before the registration fit, as described below. During the quantitative registration comparison, the RF remained attached to preserve the acquisition geometry.

**Image registration**

For a registration trial, let $f_{i}$ denote the design center of fiducial *i* in the RF coordinate system and let $x_{i}$ denote its corresponding CT coordinate (i=1,…,5). In the automatic method, $x_{i}$ is the detected marker center; in the manual method, it is the operator-selected coordinate. CT coordinates use the right–anterior–superior (RAS) convention, and transforms act on column vectors. A bar denotes homogeneous coordinates, and π extracts the three spatial components after transformation. The RF-to-CT rigid fit is defined by the least-squares objective [21]:

(1) $$T_{CT}^{RF}=\underset{T\in SE(3)}{\mathrm{arg min}}\sum_{i=1}^{5}{∥x_{i}-\pi \left(T{\bar{f}}_{i}\right)∥}^{2}$$

where SE(3) denotes rigid rotations and translations without scaling.

**Coordinate handover and implemented fit**

While both frames are visible, their tracked poses define the RF-to-DRF relation:

(2) $$T_{DRF}^{RF}={\left(T_{OTS}^{DRF}\right)}^{-1}T_{OTS}^{RF},\;T_{CT}^{DRF}=T_{CT}^{RF}{\left(T_{DRF}^{RF}\right)}^{-1}.$$

The implementation first obtains $T_{DRF}^{RF}$, expresses the five design fiducials in DRF coordinates as $\pi (T_{DRF}^{RF}{\bar{f}}_{i})$, and fits these points to the five CT coordinates to obtain the actual registration matrix $T_{CT}^{DRF}$. For the same correspondences and rigid $T_{DRF}^{RF}$, the resulting matrix $T_{CT}^{DRF}$ equals $T_{CT}^{RF}{\left(T_{DRF}^{RF}\right)}^{-1}$ within numerical precision. Both methods use the same rigid registration procedure. The matrices $T_{DRF}^{RF}$ and $T_{CT}^{DRF}$ are saved for each trial; $T_{DRF}^{RF}$ is held fixed within that trial, and is not assumed to be numerically identical across trials.

**Instrument projection during navigation**

The stored $T_{CT}^{DRF}$ maps DRF coordinates to CT coordinates. The live tool pose and DRF pose are supplied independently by the OTS. A calibrated tool-tip point ${\bar{P}}_{Tip}$, expressed in homogeneous tool coordinates, is displayed in CT space as

(3) $$P_{CT}\left(t\right)=\pi \;\left[T_{CT}^{DRF}\;{\left(T_{OTS}^{DRF}\left(t\right)\right)}^{-1}T_{OTS}^{Tool}\left(t\right){\bar{P}}_{Tip}\right].$$

This expression uses the current DRF and instrument poses without a new RF input. Tool-tip calibration, such as pivot calibration, supports this navigation function; a tracked tool is not used in the center-based accuracy evaluation below. The RF must remain fixed relative to the target anatomy from CT acquisition until handover, and the DRF must remain rigidly coupled to that anatomy thereafter.

### 2.3. Automatic Marker Detection and Registration Algorithm

Automatic marker detection in this study used peak-relative thresholding followed by connected-component extraction, geometric filtering, and fiducial identification using the RF design geometry. The identified CT fiducials and the tracked RF-to-DRF relationship were used to compute the common DRF-to-CT rigid registration described in Section 2.2.

**Figure 3:**
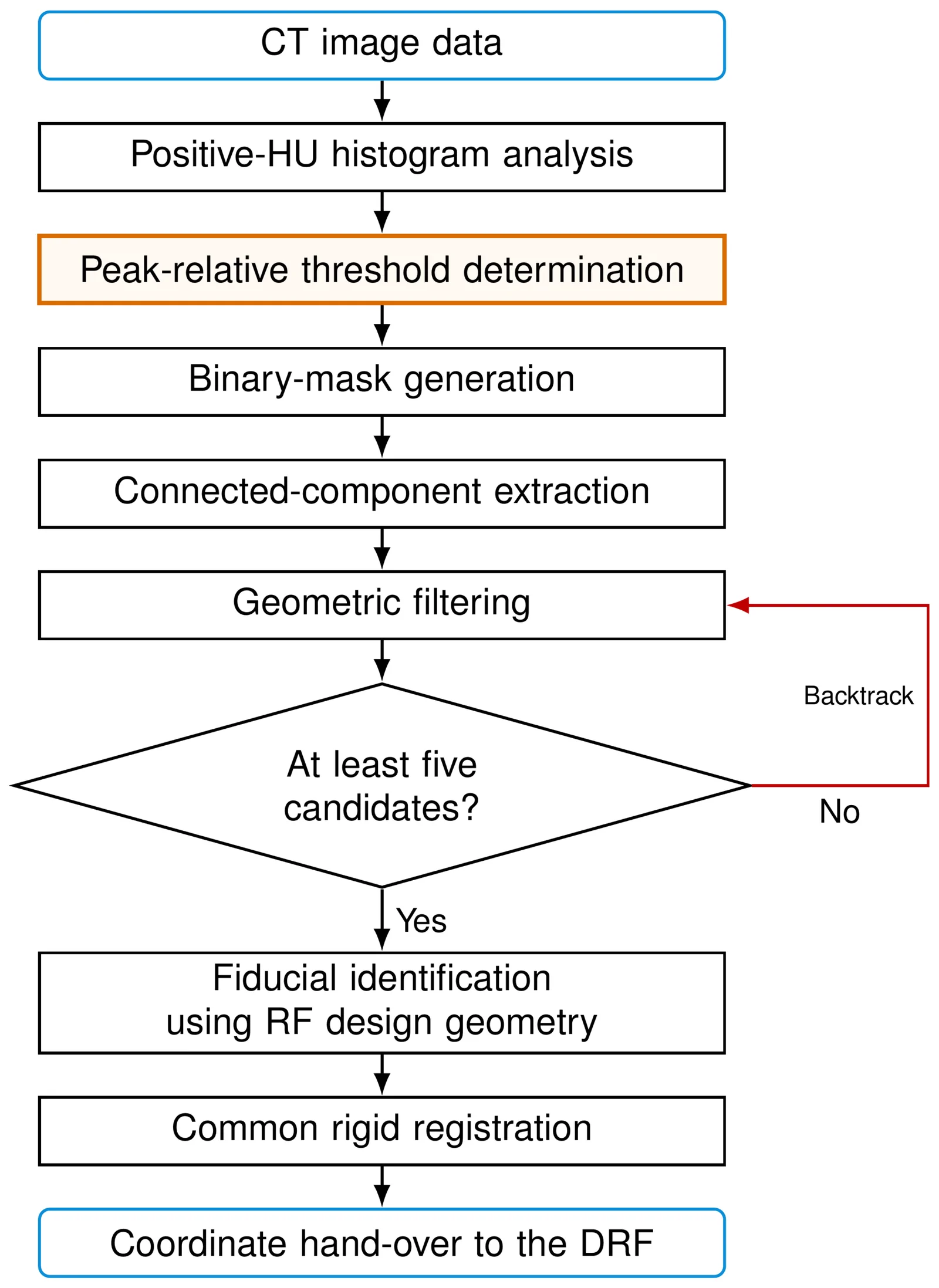
Automatic marker detection and registration using peak-relative threshold determination (orange) and the RF design geometry, with backtracking for candidate selection and coordinate handover to the DRF.

Figure 3 summarizes the registration-fiducial detection path; the same detector is invoked in three-target mode for evaluation.

The detector computes a 1500-bin histogram from positive-HU voxels, smooths it with a five-bin moving average, and searches for the dominant peak at HU ≥100. The threshold is selected at the first subsequent bin at or below 1% of the peak height and restricted to 100–4500 HU. Thresholding is followed by 26-neighbor connected-component extraction. Candidate centers are binary-component centroids. The voxel count range is derived from the nominal 2.3-mm sphere volume and voxel volume: from max(⌊0.6N⌋,20) to ⌊3.0N⌋, where *N* is the expected sphere voxel count. Subsequent filters use solidity ≥0.4, sphericity ≥0.4, elongation ≤1.6, and design geometry constraints.

The final registration step requires five geometrically valid fiducials, and selects the correspondence with the lowest RMS error from a proper rigid fit. Successful completion requires an unambiguous correspondence, a fresh RF–DRF tracking snapshot, and a newly generated finite proper rigid transformation.

For target evaluation, three centers are required and their six possible correspondences are compared against the fixed five-fiducial prediction. Ambiguous target correspondence is flagged for review.

The Supplementary Materials include saved-coordinate replay using the numerical core and the actual recorded RF-to-DRF and DRF-to-CT transforms, together with analysis scripts. The acquired CT volumes and complete real-time UI and tracking SDK are not included; saved-coordinate replay does not repeat image detection or hardware timing.

### 2.4. Accuracy Evaluation

**Figure 4:**
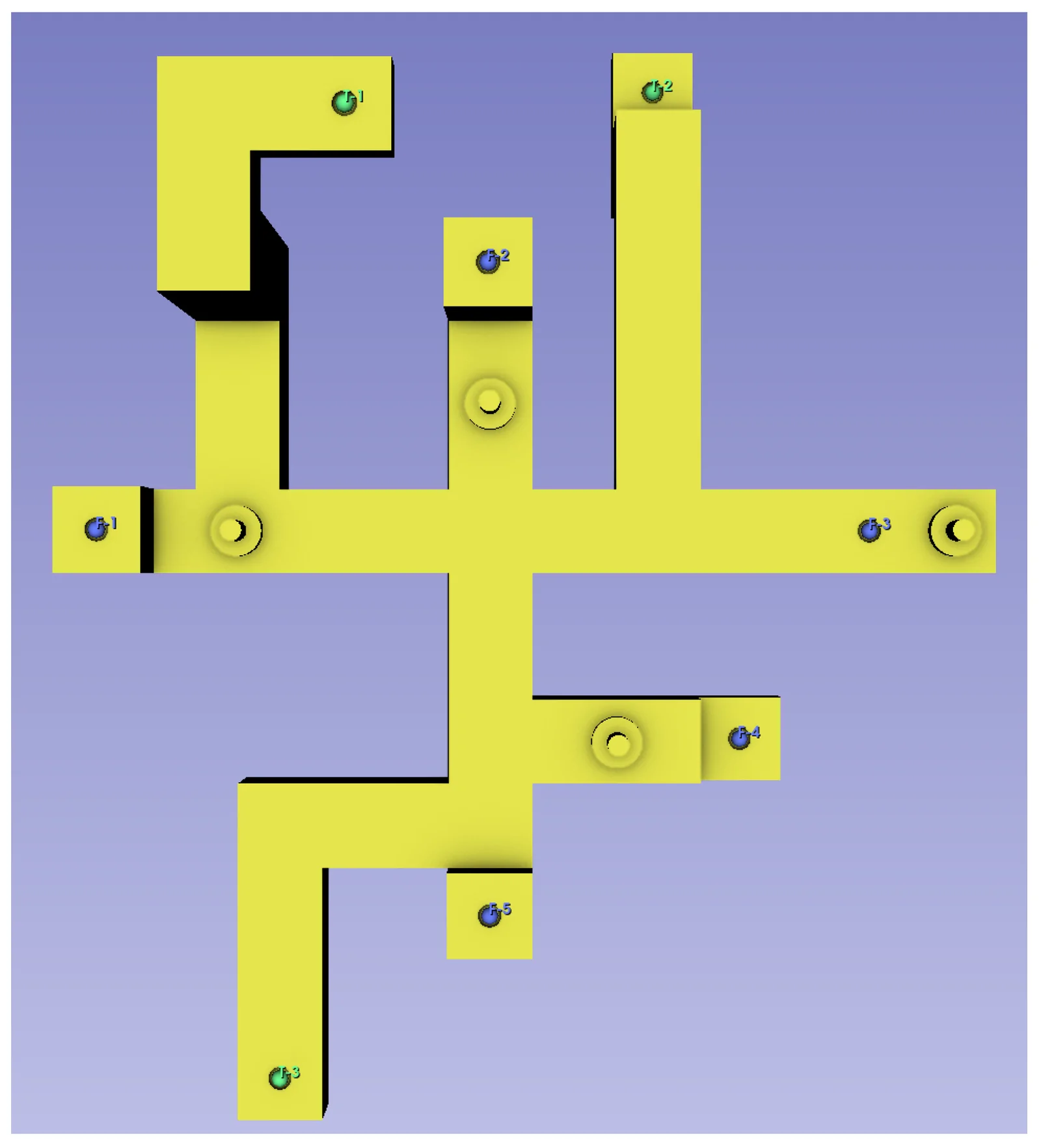
Functional grouping of RF markers for accuracy evaluation: CT markers used for fiducial registration error (FRE) computation (blue, F-1 to F-5) and CT markers used as target registration error (TRE) targets (green, T-1 to T-3).

The fiducial registration error (FRE) describes residual alignment at the points used for registration, whereas the target registration error (TRE) is evaluated at corresponding points excluded from transform estimation [19]. Both methods were evaluated with the same five registration fiducials and the same three on-frame target centers identified in Figure 4. TRE was defined as the root mean square error (RMSE) between transformed design centers and detected CT centers. This common center-based definition avoids probe surface contact and tip calibration contributions to the comparison.

**Fiducial registration error**

Using the trial’s $T_{DRF}^{RF}$ and actual $T_{CT}^{DRF}$ defined above, the FRE is calculated from the five paired fiducials as follows:

(4) $${\mathrm{FRE}}_{\mathrm{RMSE}}=\sqrt{\frac{1}{5}\sum_{i=1}^{5}{∥x_{i}-\pi \left(T_{CT}^{DRF}T_{DRF}^{RF}{\bar{f}}_{i}\right)∥}^{2}}.$$

**On-frame design-referenced target registration error**

Let $r_{j}$ be target *j*’s design center in RF coordinates and $c_{j}$ its detected CT center (j=1,2,3). Applying the saved $T_{DRF}^{RF}$ and $T_{CT}^{DRF}$ expresses the design center in CT coordinates. The target distances and their trial-level RMSE are

(5) $$e_{j}=∥c_{j}-\pi \left(T_{CT}^{DRF}T_{DRF}^{RF}{\bar{r}}_{j}\right)∥,\;{\mathrm{TRE}}_{\mathrm{RMSE}}=\sqrt{\frac{1}{3}\sum_{j=1}^{3}e_{j}^{2}}.$$

All norms are Euclidean distances between three-dimensional positions, in millimeters. Target candidates in CT are screened using their three-point design geometry; correspondences are assigned using the fixed five-fiducial RF-to-CT fit $T_{CT}^{RF}$, without re-estimating $T_{CT}^{DRF}$ from the target points. The same target detection procedure and settings provide the CT reference centers for both registration methods.

The common evaluation retains the trial’s actual registration matrix and distinguishes the contribution of fiducial coordinate determination under a shared center-based measurement protocol. With the same rigid $T_{DRF}^{RF}$ applied to registration and evaluation points, $T_{CT}^{DRF}T_{DRF}^{RF}$ equals the direct RF-to-CT fit $T_{CT}^{RF}$ within numerical precision. The endpoint assesses agreement between withheld design centers and CT centers; it is not an independent measurement of optical tracking or handover accuracy. Fabrication deviations and CT-center localization can contribute to the discrepancy.

### 2.5. Experimental Design

**Figure 5:**
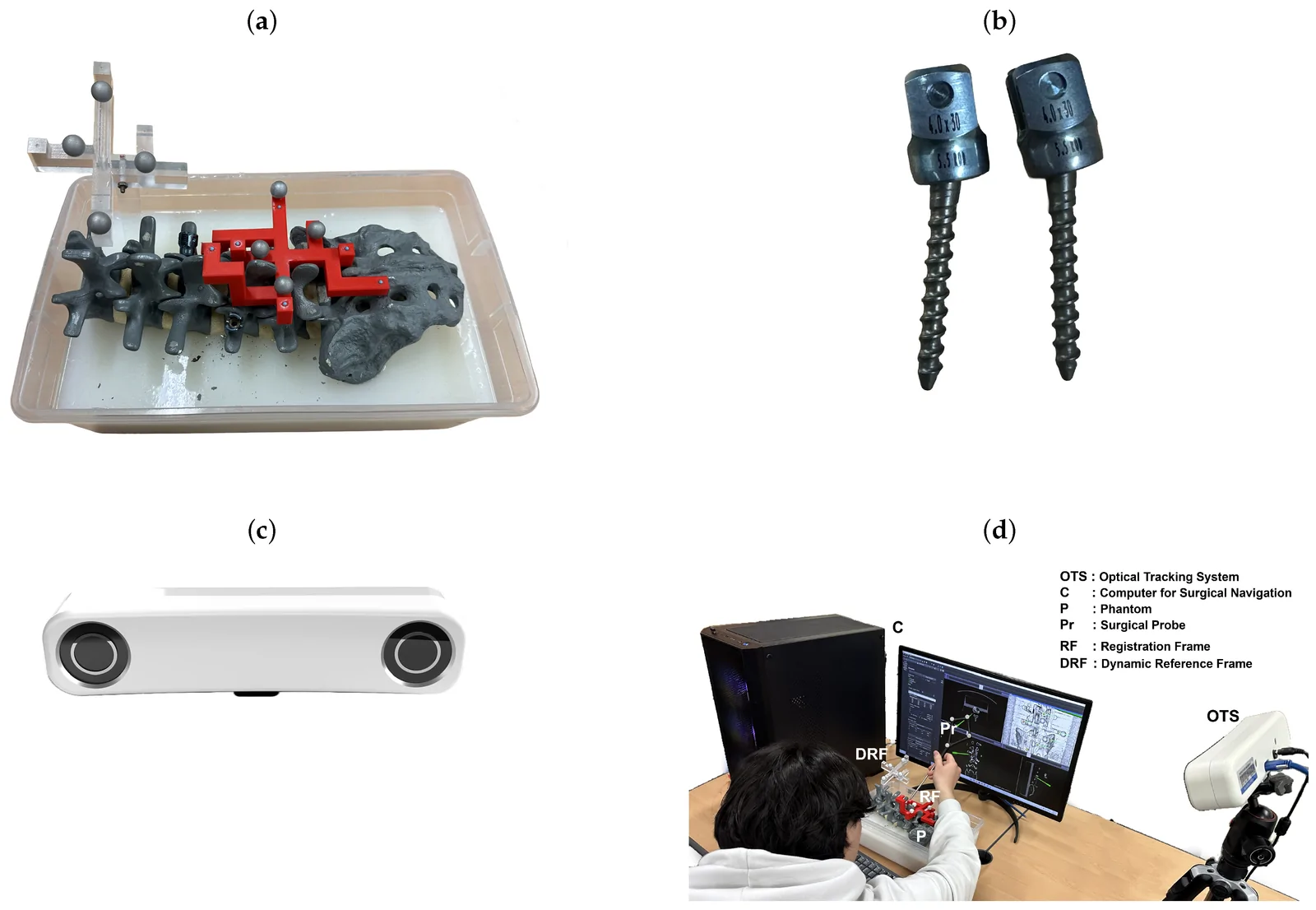
Fixed phantom setup for the center-based evaluation. (**a**) RF (red) attached to the target vertebra and DRF (silver), mounted on a rod in the same fixed phantom assembly. The lower phantom portion was embedded in silicone, with the relative configuration preserved from CT acquisition through repeated registrations. (**b**) Pedicle screws in L3 (right) and L4 (left), representing metallic implants. (**c**) Optical tracking system. (**d**) Overall navigation environment, where the tracked probe was used for visual checks of tool tracking and its display on the navigation monitor. The center-based FRE/TRE evaluation did not use a tracked probe.

Automatic and manual fiducial coordinate determination were evaluated using a synthetic lumbar sawbone phantom in a fixed configuration (Figure 5). The setup included metallic pedicle screws and preserved the CT acquisition geometry for repeated registrations; soft tissue motion and RF retrieval were outside this quantitative comparison.

- **Base fixation:** Polystyrene foam blocks were placed inside a plastic container and a lumbar sawbone phantom was positioned on top. Silicone was poured to a level that submerged the lower portion of the phantom, providing rigid fixation once it had cured.
- **Pedicle screw insertion:** Two titanium pedicle screws were inserted into the sawbone phantom (L3 right and L4 left pedicles) to simulate the presence of metallic implants in the surgical field (Figure 5b).
- **DRF installation:** A rod-type support was inserted into the polystyrene foam block adjacent to the sawbone phantom, and the DRF was mounted on top. The support formed part of the fixed phantom assembly and maintained the DRF pose relative to the phantom.
- **Hybrid marker attachment:** The hybrid marker (RF) was placed on the target vertebra of the sawbone phantom and rigidly fixed with adhesive.

The CT acquisition and subsequent navigation experiments used this fixed phantom–RF–DRF assembly. The primary purpose of fixation was to preserve the relative positions and orientations present during CT acquisition so that repeated registrations could use the same CT dataset. The support placement was also intended to reduce obstruction of RF tracking by the DRF.

Real-time marker tracking was performed with a stereo-camera optical tracking system (SKADI, DigiTrack) with a manufacturer-specified positional accuracy of 0.2 mm RMS at a frame rate of 50 fps, operating at a wavelength of 850 nm with a measurement range of 500–1500 mm. The navigation experiments were performed using 3D Slicer 5.8.1 with custom registration and tracking modules.

Three fixed CT datasets, one from each imaging device summarized in Table 1, were used to compare the methods under these acquisition conditions. The C-arm CT device (Genoray) is a pre-commercial prototype system.

The comparison used the three CT datasets with the following common protocol:

- **Registration inputs:** Automatic registration used the detected centers of F-1 to F-5. In manual registration, ten operators located the same five fiducials in CT and placed registration points using the navigation interface. They were instructed to work as quickly and accurately as possible, reflecting the speed and accuracy demands of navigation workflows. Operator-selected coordinates were preserved and ordered for correspondence. Operators had received interface training and provided written informed consent under the approved protocol (see Institutional Review Board Statement). Each operator contributed five trials per CT to the analysis set, making for 150 manual trials. Each block of five trials used the same CT, and actual CT block order was recorded. Both methods used the same experimental computer, software version, settings, and rigid registration procedure.
- **Registration and evaluation:** The five design fiducials were expressed in DRF coordinates using the trial’s tracked relation $T_{DRF}^{RF}$ and registered to their CT counterparts to produce $T_{CT}^{DRF}$. The RF remained attached. FRE and the common three-target center-based TRE were calculated using the saved $T_{DRF}^{RF}$ and actual $T_{CT}^{DRF}$. This calculation did not require new RF input after registration or contact with a probe. For each fixed CT, one automatic result was used as the comparison value; repeated software execution was not counted as a new CT acquisition. Raw coordinates, matrices, settings, and trial records were saved.
- **Processing time:** Timing began after the common CT/design/logging checks had been completed and the attempt start record had been saved, and ended when the registration function returned and a new DRF-to-CT registration matrix was confirmed. The automatic interval included CT processing and five-fiducial detection; the manual interval included identifying and selecting those five fiducials. Both included live tracking acquisition and registration. Within-trial recovery, waiting, and issue recording time remained included. Post-registration display handling, target detection and TRE calculation, and final result file writing were excluded. RF placement, CT acquisition, prior transfer/loading, separate verification, and removal were outside this software processing interval.

The RF remained attached throughout the quantitative registration comparison. A separate earlier functional check examined whether navigation could continue when RF input was unavailable by physically obscuring its optical markers while preserving the phantom geometry; this check addresses dependence on RF input, but does not quantify the mechanical effects of retrieval.

### 2.6. Study Sample

The study used a ten-operator design with five manual registrations per operator and CT designated for analysis, alongside one automatic benchmark for each of the three existing CT datasets. Operators were treated as the independent sampling units for the conditional method comparison, as opposed to repeated trials or the three evaluation targets. The sample size was not based on a prospective power calculation for the center-based TRE endpoint.

### 2.7. Statistical Analysis

The primary outcome was the three-target TRE RMSE. For operator *p* and fixed CT dataset *k*, the five manual trial values were averaged to obtain ${\bar{y}}_{pk}$. With the observed automatic value $a_{k}$ held fixed, the operator-specific contrast was

(6) $$D_{p}=\frac{1}{3}\sum_{k=1}^{3}\left({\bar{y}}_{pk}-a_{k}\right),\;\hat{\Delta }=\frac{1}{10}\sum_{p=1}^{10}D_{p},$$

with positive values indicating lower TRE for the automatic benchmark. The primary comparison was $\hat{\Delta }$, with the three CT datasets equally weighted. Operators were resampled with replacement in 10,000 bootstrap replicates, retaining each operator’s CT datasets and repeated trials together. The 2.5th and 97.5th percentiles of the replicate mean differences formed the 95% percentile confidence interval (CI). This directly summarizes the operator resampling distribution while preserving within-operator dependence. The stored resampling indices were generated using NumPy PCG64 with seed 20261002, and percentiles were calculated using linear interpolation; automatic benchmarks were not resampled.

For the same overall TRE mean difference, a *t*-based sensitivity interval was calculated as $\hat{\Delta }\pm t_{0.975,9}s_{D}/\sqrt{10}$, where $s_{D}$ is the sample SD of the ten $D_{p}$ values. Individual $D_{p}$ values were retained to show their distribution. CIs quantify uncertainty in the estimated mean difference, conditional on the three CT datasets, tested frame, settings, and observed automatic benchmarks; they are distinct from the between-operator dispersion and automatic repeat variability. SDs were identified separately for trial values and operator means. Both the CT-specific CIs and those for the secondary FRE and processing time outcomes are pointwise descriptive summaries, without adjustment for simultaneous coverage. The primary analysis focused on effect estimation and CIs, and did not include hypothesis tests.

Manual fiducial coordinate dispersion was summarized by the RMS distance from each fiducial’s mean selected location, with the within-operator and between-operator components described separately. The CT-specific RMS summary pooled squared distances across the five fiducials and 50 trials (250 coordinates) before taking the square root, so it does not represent an arithmetic mean of the five fiducial-specific RMS values. Distances to the corresponding automatic CT centers characterized differences in selected coordinates, using those centers as comparison points rather than as independently measured marker positions.

## 3. Results

### 3.1. Fabrication Check

The inspected frame dimensions, thicknesses, and center-to-center marker distances differed from their design values by approximately 0.04–0.15 mm.

### 3.2. Automatic Marker Detection Results

In the three recorded automatic runs, the five registration fiducials and three evaluation targets were detected using peak-relative thresholding. The thresholds were 2830.16, 423.64, and 796.13 HU for C-arm CT, conventional CT, and PET/CT, respectively. No numerically ambiguous correspondence was flagged, and no manual intervention was required in these three runs.

**Figure 6:**
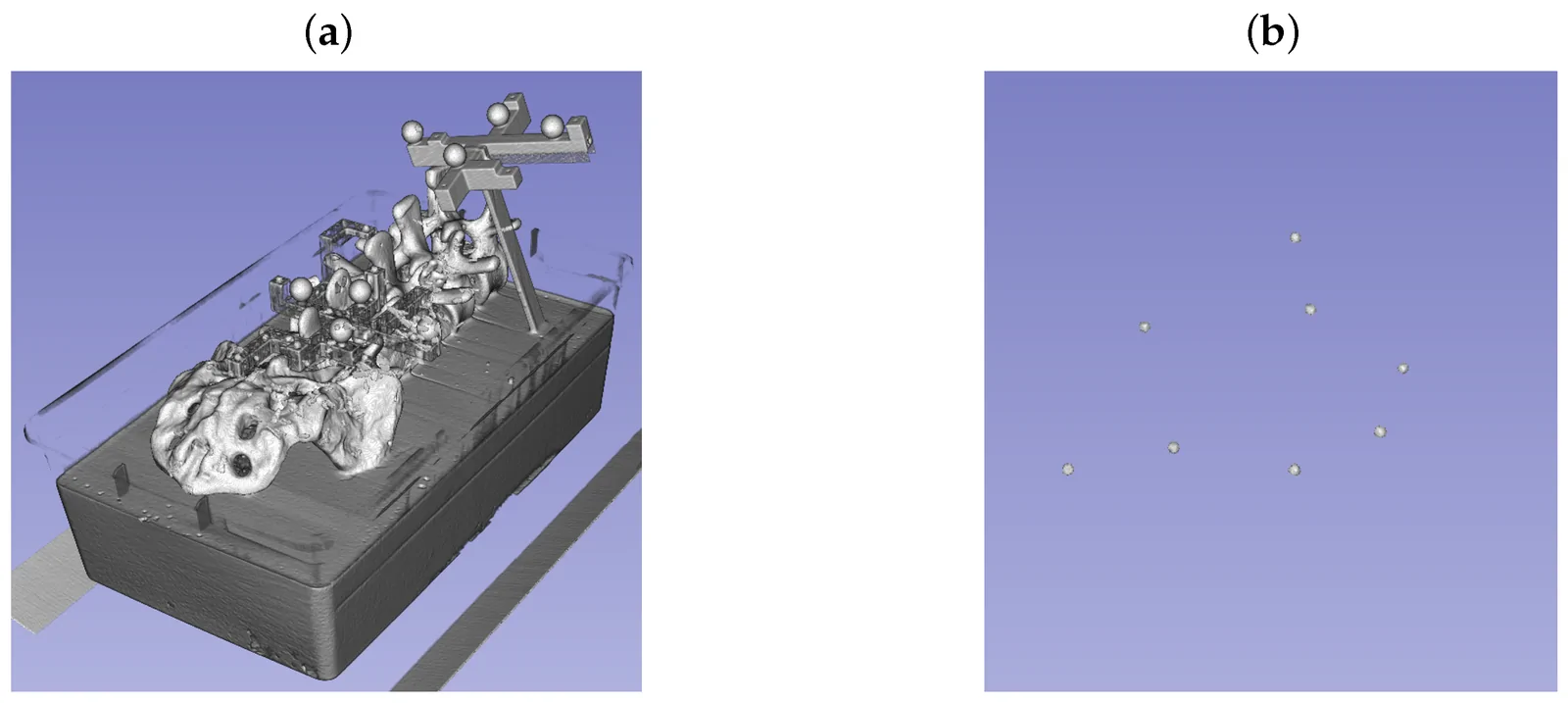
Visualization of the filtering pipeline result: (**a**) 3D volume rendering of the original C-arm CT data, showing the hybrid marker and surrounding structures; (**b**) final identified CT markers after thresholding and geometric filtering.

**Figure 7:**
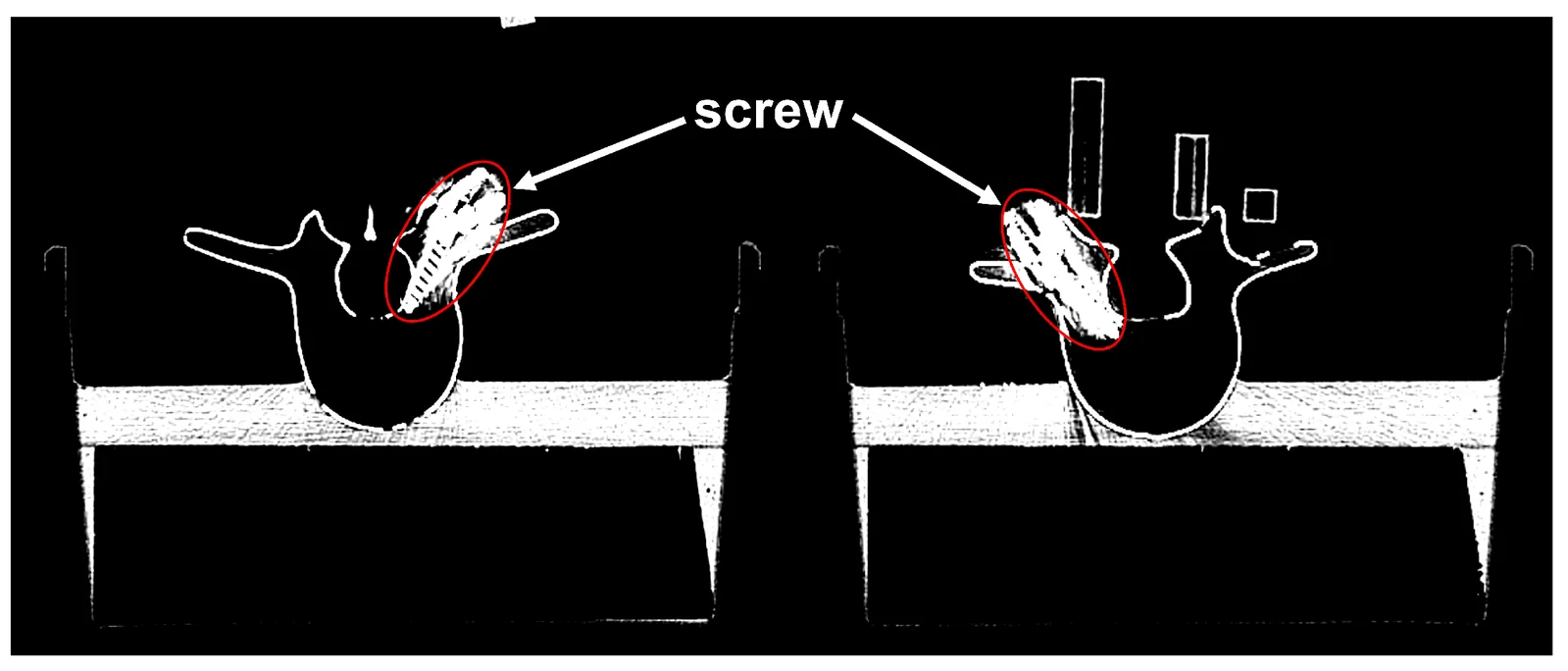
PET/CT slice images showing the two pedicle screws in the sawbone phantom used to evaluate marker detection in the presence of metallic artifacts.

Figure 6 illustrates candidate extraction in C-arm CT. The phantom contained two pedicle screws (L3 right and L4 left; Figure 5b), also visible in the PET/CT slices in Figure 7.

### 3.3. Registration Outcomes

The analysis comprised 150 manual registrations (five per operator and CT) and one automatic benchmark per CT. Table 2 reports the FRE, TRE, and processing time. The automatic on-frame TRE values were 0.486, 0.546, and 0.581 mm, compared with manual trial means of 1.524, 1.625, and 1.545 mm for C-arm CT, conventional CT, and PET/CT, respectively, while the equally weighted means were 0.538 and 1.565 mm. No repeat variability estimate was assigned to the single automatic observations.

The earlier RF occlusion check confirmed continued DRF-based navigation when RF input was unavailable.

### 3.4. Operator-Level Mean Differences

The primary overall manual-minus-automatic TRE difference was 1.027 mm (95% percentile operator-bootstrap CI, 0.951–1.148 mm; see Table 3). The $t_{9}$ sensitivity interval for the same mean was 0.900–1.154 mm. Individual operator contrasts ranged from 0.921 to 1.512 mm, with a sample SD of 0.178 mm; all ten were positive (see Supplementary Table S1).

Mean FRE across equally weighted CT datasets was 0.149 mm for the automatic method and 0.679 mm for manual registration. The corresponding mean difference was 0.530 mm (pointwise 95% CI, 0.355–0.788 mm). Mean processing times were 2.817 and 22.589 s, with a difference of 19.773 s (pointwise 95% CI, 17.308–22.513 s).

**Figure 8:**
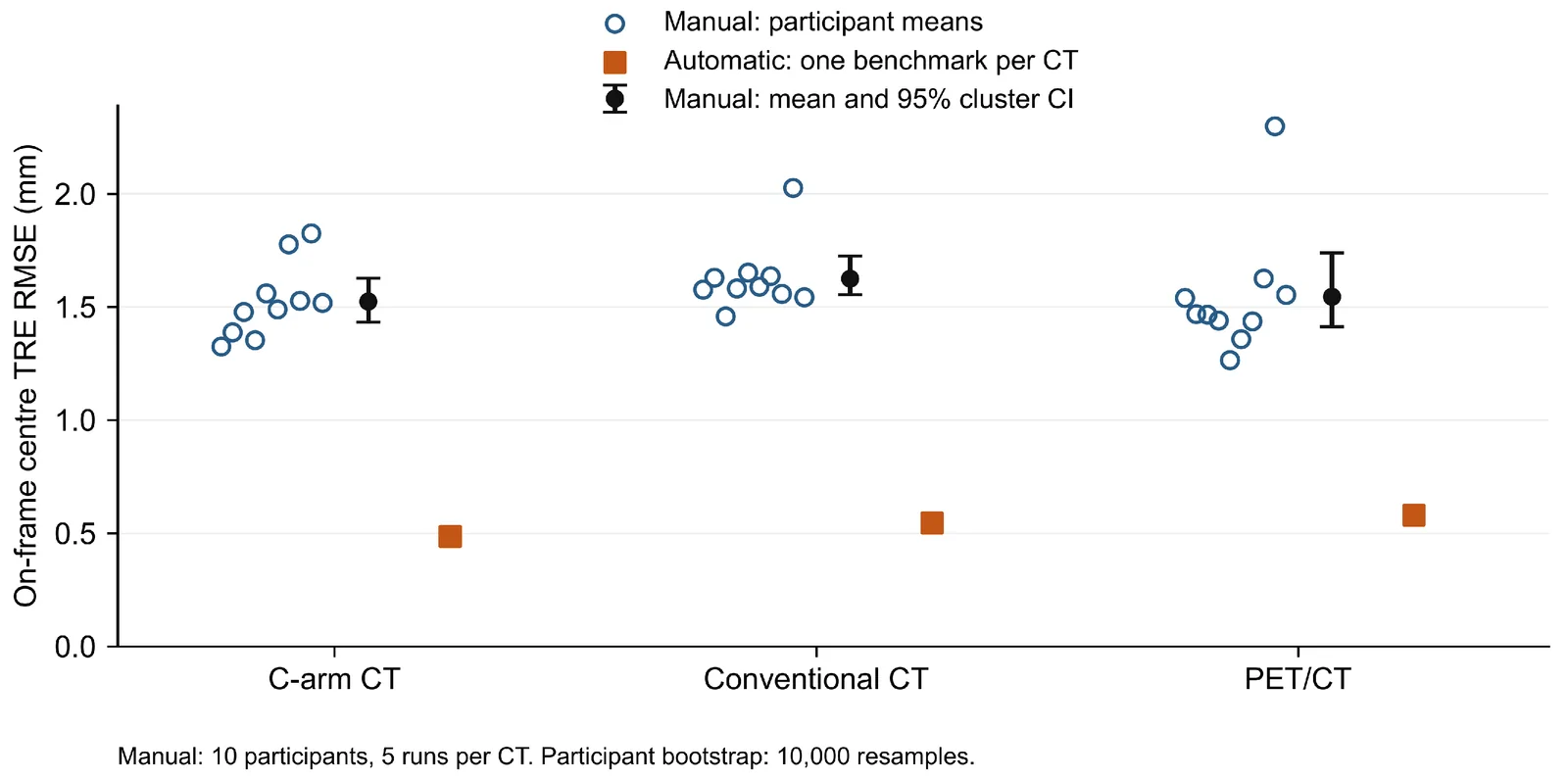
On-frame center TRE by fixed CT dataset. Open circles represent each operator’s five-trial mean (n=10); black points and bars represent the manual mean and pointwise 95% percentile operator-bootstrap CI. Orange squares are the single observed automatic benchmarks, without automatic SD or CI. Error bars describe uncertainty in the mean, not operator dispersion. These CT-specific summaries are secondary to the overall contrast.

**Figure 9:**
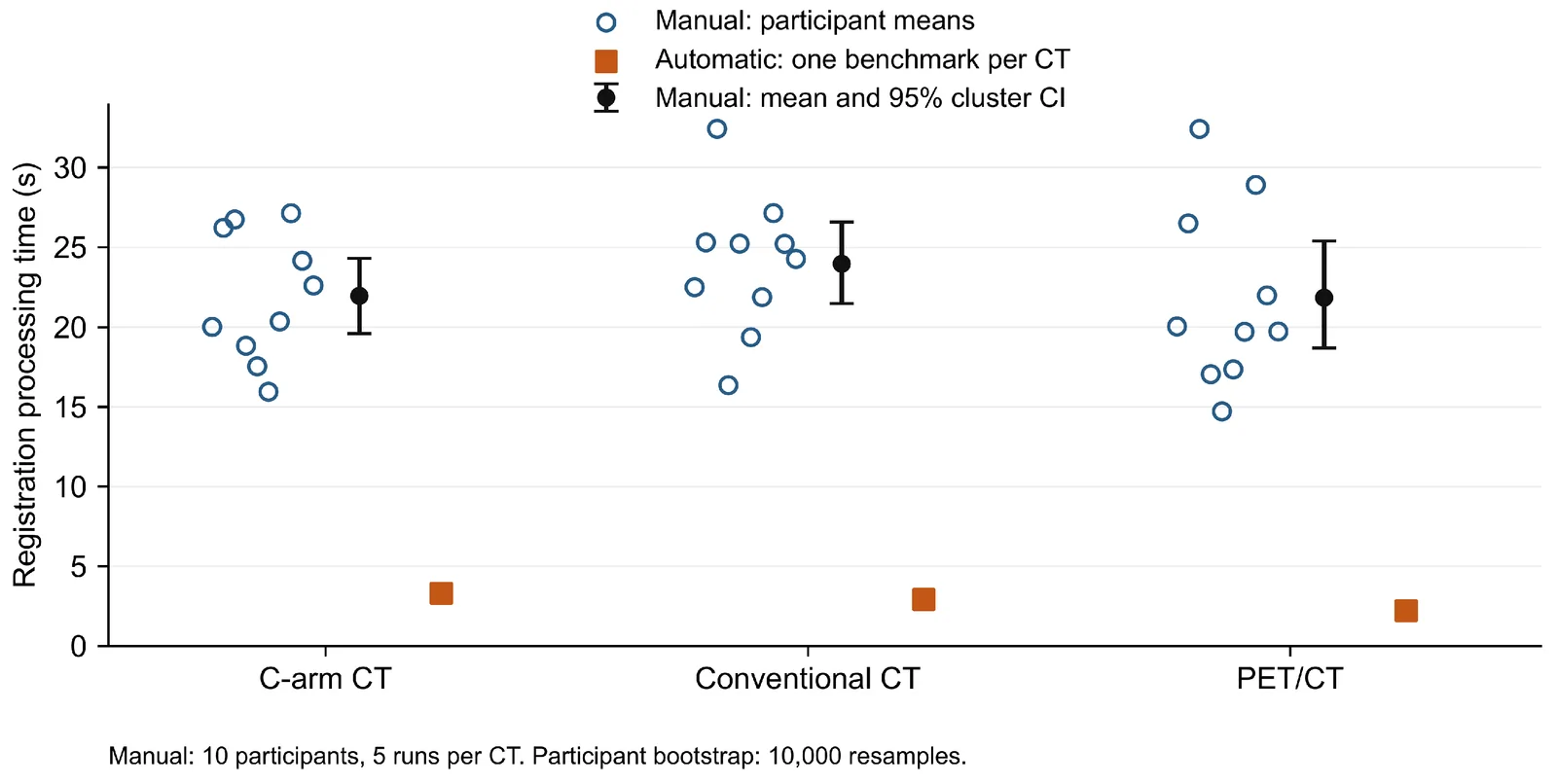
Registration processing time by fixed CT dataset. Open circles represent each operator’s five-trial mean; black points and bars represent the manual mean and pointwise 95% percentile operator-bootstrap CI. Orange squares are single automatic executions. The measured registration interval includes registration and handover while excluding subsequent TRE evaluation and the imaging workflow.

Figure 8 shows the CT-specific operator means and fixed automatic TRE benchmarks, while Figure 9 shows processing times.

**Figure 10:**
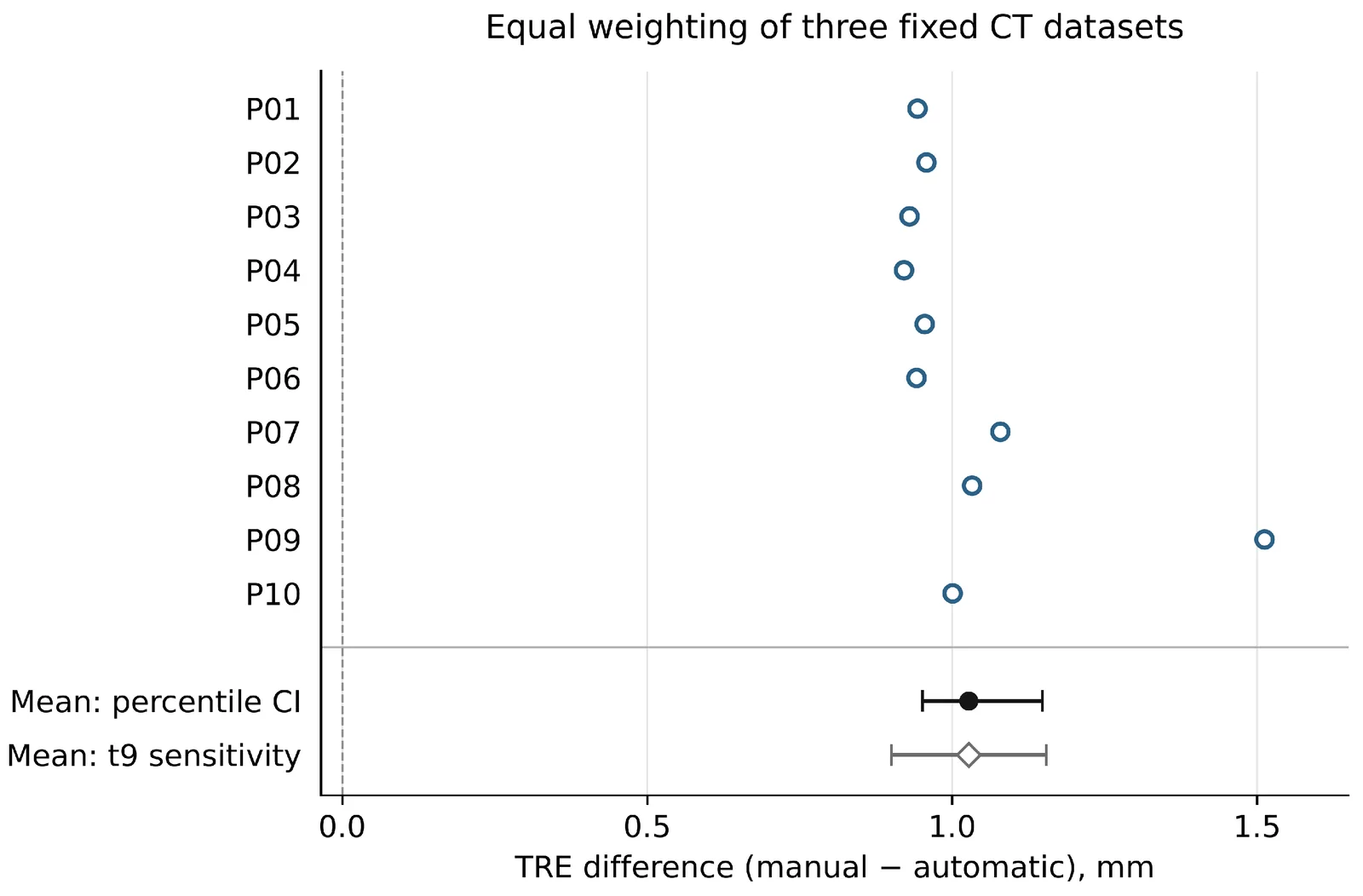
Individual operator contrasts and uncertainty in the overall mean TRE difference. Positive values indicate lower TRE for the automatic benchmark. Each open circle is $D_{p}$, the equally weighted mean of three CT-specific manual-minus-automatic differences. The black point and bar show the overall mean with its 95% percentile bootstrap CI; the diamond and bar show the same mean with its $t_{9}$ sensitivity interval. Automatic observations are fixed. The intervals estimate uncertainty in the same mean, and are not ranges of operator dispersion.

Figure 10 displays the ten overall operator contrasts and the two intervals for their mean.

### 3.5. Selected Fiducial Coordinates

The RMS scatter of manual coordinates around each fiducial’s mean selected position was 1.091, 1.065, and 1.120 mm for C-arm CT, conventional CT, and PET/CT, respectively, while the RMS distances from the manual coordinates to the corresponding automatic CT centers were 1.729, 1.817, and 1.709 mm in the same order. The automatic centers served as comparison points rather than independently measured marker positions. CT-specific summaries and individual fiducial dispersion are provided in the Supplementary Materials (Table S2 and the accompanying coordinate tables).

## 4. Discussion

The integrated workflow produced lower on-frame center TRE and shorter registration processing times than the observed manual point selection procedure in the three fixed CT datasets. The equally-weighted TRE difference was 1.027 mm (95% operator-bootstrap CI, 0.951–1.148 mm), and the processing time difference was 19.773 s. Using the same CT datasets, fiducial identities, rigid registration procedure, and evaluation centers allowed the comparison to focus on how the fiducial coordinates were determined.

The coordinate analysis helps to interpret the TRE difference: manual selections varied across repeated observations and differed from the automatically detected centers. During data collection, the investigator observed that many operators selected locations near the visible upper marker surfaces; therefore, the comparison reflects the point selection practices adopted under instructions to work quickly and accurately, including both selection variability and positional differences from the automatic centers. Performance under a separate manual center-refinement protocol was not evaluated.

Automatic detection returned deterministic centers for a fixed CT and settings; elapsed time and the DRF-referenced transform could still vary with runtime conditions and the measured RF–DRF relation. Because the comparison included one automatic result per CT, these observations were treated as fixed benchmarks. Peak-relative thresholding detected the required markers in all three recorded automatic runs of the screw-containing phantom, supporting this detection path under the tested imaging conditions.

To compare the methods on a common basis, the evaluation used corresponding design and CT centers, excluding probe surface contact geometry and tool-tip calibration from the endpoint. The three evaluation centers were withheld from the registration fit, so their TRE assesses agreement beyond the fitted fiducials; FRE alone cannot establish target accuracy [19]. Because these targets remain on the RF and the shared rigid relation cancels in the composite $T_{CT}^{DRF}T_{DRF}^{RF}$, the endpoint measures on-frame center agreement rather than the complete error of an independently tracked instrument at an anatomical target. Fabrication deviations and CT center localization can contribute to this discrepancy. Caliper inspection characterized deviations in selected fabrication dimensions, and optical marker registration defined the origin and axes connecting the design coordinates to tracking. Together, these procedures document the fabrication dimensions and coordinate definition underlying the evaluation; optical calibration uncertainty and accuracy at independent anatomical targets require separate assessment.

The statistical analysis likewise follows the comparison design. Resampling the ten operators with their repeated observations preserved within-operator dependence and estimated uncertainty in the mean difference without treating 150 trials as independent operators. The individual operator contrasts show variation among operators, while the $t_{9}$ sensitivity interval provides an alternative estimate of uncertainty in their mean difference. Inference remains conditional on the three CT datasets, frame, settings, and fixed automatic benchmarks; the three volumes represent different acquisition conditions rather than repeated independent acquisition cycles.

Published navigation results provide context, but their target definitions and measurement protocols differ from the present on-frame evaluation [14]. The closest workflow comparison is that of Frisk et al., who compared surface matching on preoperative CT with automatic registration on intraoperative CBCT using Universal AIR [16]. Their dual-modal registration matrix could rest on the skin beside the incision; registration was transferred to a separate DRF clamped to an exposed spinous process, allowing the matrix to be removed. The present workflow shares this handover principle. Its contribution is the asymmetric RF implementation, integration of design geometry-based marker identification with rigid registration and handover, and evaluation of automatic coordinate determination under common conditions. In contrast to Frisk et al.’s comparison of registration approaches and imaging datasets, the present experiment kept the CT dataset, fiducials, final registration procedure, and evaluation centers common. Their screw head measurements with a tracked pointer and our on-frame center measurements address different endpoints, so the reported TRE values do not provide a direct ranking of the systems.

The separation of registration from subsequent tracking is central to the proposed clinical use. The RF must retain its relationship to the target anatomy from CT acquisition through handover. Once handover is complete, the stored DRF-to-CT registration and live DRF and tool poses support navigation without further RF input. Motion shared by the DRF and target vertebra is represented by live tracking, whereas motion of the RF or surrounding skin after retrieval no longer changes the tracking reference. Changes in DRF-to-bone fixation or relative motion between vertebral levels require reassessment because the tracked DRF alone does not represent those changes. The fixed phantom–RF–DRF assembly preserved the acquisition geometry for the quantitative comparison; complete RF retrieval, repositioning and re-imaging cycles were not evaluated. Validation of those cycles would require independent anatomical targets and verification of DRF-to-anatomy coupling after manipulation. The rigid phantom and trained non-clinical operators also differ from the soft tissue conditions and users of an operative setting.

The proposed re-registration workflow places the RF near each new target to address distance-related error [19]. Clinical use of a more proximal reference arc for long multi-level instrumentations provides a related rationale [20]. At a new target, the RF can be placed temporarily on a supporting surface, then a new CT can be acquired and automatic registration and handover repeated. The new CT and RF-to-DRF relationship incorporate the new placement, so the RF need not reproduce its previous position and orientation. Lifting away the RF avoids the fixation release actions associated with adhesive, clamps, or screws, while the DRF remains rigidly coupled to the target anatomy. Because the updated registration depends on the maintained DRF-to-anatomy relationship, the proposed sequence includes checking DRF fixation and correspondence between physical and displayed positions after registration. The measured automatic processing interval of 2.817 s supports rapid registration and handover after image loading. CT acquisition and transfer time, positional verification, and radiation exposure remain additional considerations when assessing the complete clinical workflow.

## 5. Conclusions

The asymmetric dual-modal RF integrates design geometry-based marker identification, rigid registration, and coordinate handover to a separate DRF. In a common center-based comparison using three fixed CT datasets, automatic registration yielded a mean on-frame TRE of 0.538 mm compared with 1.565 mm for the observed manual selection procedure, a difference of 1.027 mm (95% operator-bootstrap CI, 0.951–1.148 mm). Mean registration processing times were 2.817 and 22.589 s, respectively. These results demonstrate the benefit of automatic fiducial determination for on-frame center agreement and processing time under the tested conditions, providing a basis for further validation of anatomical accuracy and repeated target-proximal registration.

## Figures and Tables

**Table 1:** Specifications of the three CT imaging devices used for validation.

Specification	C-Arm CT	Conventional CT	PET/CT
Manufacturer	Genoray	SIEMENS	SIEMENS
Model	Pre-commercial prototype	SOMATOM Definition AS+	Biograph128
Tube voltage (kVp)	90	120	80
Tube current (mA)	3.0	20	64
Voxel size (mm)	0.30×0.30×0.30	0.62×0.62×0.60	0.59×0.59×0.60
Image matrix	572×572	512×512	512×512

**Table 2:** Manual and automatic registration outcomes in the three fixed CT datasets. Manual values are arithmetic means ± sample SDs of 50 trials per CT (10 operators × 5 trials); automatic values are single observed benchmarks, one per CT. FRE and TRE are per-trial RMS Euclidean distances across five registration fiducials and three on-frame targets, respectively. Processing time includes registration and handover, excluding CT acquisition, transfer/loading, and subsequent TRE evaluation.

CT Dataset	Method	FRE RMSE (mm)	TRE RMSE (mm)	Registration Time (s)
C-arm CT	Automatic	0.149	0.486	3.309
	Manual	0.672 ± 0.501	1.524 ± 0.263	21.955 ± 4.584
Conventional CT	Automatic	0.170	0.546	2.921
	Manual	0.671 ± 0.369	1.625 ± 0.234	23.971 ± 6.193
PET/CT	Automatic	0.128	0.581	2.221
	Manual	0.695 ± 0.441	1.545 ± 0.389	21.842 ± 6.147

**Table 3:** Manual-minus-automatic TRE differences and 95% confidence intervals (CIs). The primary overall difference equally weights the three fixed CT datasets. Percentile bootstrap CIs use 10,000 resamples of the ten operators, retaining each operator’s CT and trial grouping and holding automatic benchmarks fixed. They describe mean-difference uncertainty for the tested conditions; the CT-specific CIs are unadjusted pointwise summaries. The $t_{9}$ sensitivity 95% CI for the same overall mean difference is 0.900–1.154 mm. Positive differences indicate lower TRE for automatic registration. Values were rounded after calculation.

Comparison	Mean TRE Difference (mm)	Bootstrap 95% CI (mm)
Overall (primary)	1.027	0.951–1.148
C-arm CT	1.038	0.946–1.140
Conventional CT	1.080	1.008–1.180
PET/CT	0.965	0.832–1.159

## Data Availability

De-identified analyzed trial measurements, registration and target coordinates, recorded transforms, and reproducible analysis scripts are included in the Supplementary Materials. Original acquired CT volumes, identifying records, and proprietary real-time tracking components are not included.

## Supplementary Materials

The following supporting information can be downloaded at: https://www.mdpi.com/article/10.3390/s26206433/s1. The supplementary materials include a PDF presenting Tables S1–S2 and their interpretation, together with a code and data archive. The archive contains de-identified coordinates, recorded transforms and measurements for the 153 analyzed trials, saved-coordinate FRE/TRE replay, operator-level bootstrap and $t_{9}$ sensitivity calculations, and scripts regenerating Table 2 and Table 3, Tables S1–S2 and Figure 8, Figure 9 and Figure 10. Table S1 reports individual operator TRE contrasts; Table S2 summarizes manual fiducial-coordinate scatter and distances to the corresponding automatic centers. Acquired CT volumes, the real-time UI and vendor SDK are not included.

## Abbreviations

CBCT	Cone-Beam Computed Tomography
DRF	Dynamic Reference Frame
FRE	Fiducial Registration Error
HU	Hounsfield Unit
IGS	Image-Guided Surgery
OTS	Optical Tracking System
RF	Registration Frame (hybrid marker)
TRE	Target Registration Error

## References

[1] Gertzbein S.D., Robbins S.E. (1990). Accuracy of pedicular screw placement in vivo. Spine.

[2] Rampersaud Y.R., Foley K.T., Shen A.C., Williams S., Solomito M. (2000). Radiation exposure to the spine surgeon during fluoroscopically assisted pedicle screw insertion. Spine.

[3] Gelalis I.D., Paschos N.K., Pakos E.E., Politis A.N., Arnaoutoglou C.M., Karageorgos A.C., Ploumis A., Xenakis T.A. (2012). Accuracy of pedicle screw placement: A systematic review of prospective in vivo studies comparing free hand, fluoroscopy guidance and navigation techniques. Eur. Spine J..

[4] Tian N.F., Huang Q.S., Zhou P., Zhou Y., Wu R.K., Lou Y., Xu H.Z. (2011). Pedicle screw insertion accuracy with different assisted methods: A systematic review and meta-analysis of comparative studies. Eur. Spine J..

[5] Holly L.T., Foley K.T. (2003). Intraoperative spinal navigation. Spine.

[6] Helm P.A., Teichman R., Hartmann S.L., Simon D. (2015). Spinal Navigation and Imaging: History, Trends, and Future. IEEE Trans. Med. Imaging.

[7] Naik A., MacInnis B.R., Shaffer A., Krist D.T., Smith A.D., Garst J.R., Hassaneen W., Arnold P.M. (2023). Trends in Technology for Pedicle Screw Placement: A Temporal Meta-Analysis. Spine.

[8] Overley S.C., Cho S.K., Mehta A.I., Arnold P.M. (2017). Navigation and Robotics in Spinal Surgery: Where Are We Now?. Neurosurgery.

[9] Sharma A.K., Garcia de Oliveira R., Suvithayasiri S., Chavalparit P., Chang C.C., Kim Y.H., Fischer C.R., Lee S., Cho S., Kim J.S. (2025). The Utilization of Navigation and Emerging Technologies with Endoscopic Spine Surgery: A Narrative Review. Neurospine.

[10] Lee Y.S., Cho D.C., Kim K.T. (2024). Navigation-Guided/Robot-Assisted Spinal Surgery: A Review Article. Neurospine.

[11] Gouzoulis M.J., Seddio A.E., Winter A.D., Jabbouri S.S., Zhu J.R., Rubio D.R., Varthi A.G., Grauer J.N. (2024). Robotic-Assisted Versus Navigation-Assisted Posterior Lumbar Fusion: A National Database Study. Spine.

[12] Maurer C.R., Fitzpatrick J.M., Wang M.Y., Galloway R.L., Maciunas R.J., Allen G.S. (1997). Registration of head volume images using implantable fiducial markers. IEEE Trans. Med. Imaging.

[13] Temple H.T. (2019). CORR Insights^®^: Are Skin Fiducials Comparable to Bone Fiducials for Registration When Planning Navigation-assisted Musculoskeletal Tumor Resections in a Cadaveric Simulated Tumor Model?. Clin. Orthop. Relat. Res..

[14] Santos E.R.d.S., Viana F.R., Gonçalves P.E., Cunha Lima M.A., Lyra C.A.M., Santos B.F.d.O. (2024). Precision in Neuronavigation Systems: A Systematic Review and Meta-analysis. Arq. Bras. Neurocir..

[15] Furuse M., Ikeda N., Kawabata S., Park Y., Takeuchi K., Fukumura M., Tsuji Y., Kimura S., Kanemitsu T., Yagi R. (2023). Influence of surgical position and registration methods on clinical accuracy of navigation systems in brain tumor surgery. Sci. Rep..

[16] Frisk H., Burström G., Persson O., El-Hajj V.G., Coronado L., Hager S., Edström E., Elmi-Terander A. (2024). Automatic image registration on intraoperative CBCT compared to Surface Matching registration on preoperative CT for spinal navigation: Accuracy and workflow. Int. J. Comput. Assist. Radiol. Surg..

[17] Eckstein F.M., Zeller A.N., Neuhaus M.T., Korn P., Gellrich N.C., Zimmerer R.M., Rahlf B. (2022). Referencing for intraoperative navigation: Evaluation of human bias. J. Stomatol. Oral Maxillofac. Surg..

[18] Tang J., Thabit A., van Walsum T., Marroquim R., Benmahdjoub M. (2026). Manual Registration in AR-Assisted Surgical Navigation: A Comparative Evaluation. Int. J. Comput. Assist. Radiol. Surg..

[19] Fitzpatrick J.M., West J.B., Maurer C.R. (1998). Predicting Error in Rigid-Body Point-Based Registration. IEEE Trans. Med. Imaging.

[20] Scheufler K.M., Franke J., Eckardt A., Dohmen H. (2011). Accuracy of Image-Guided Pedicle Screw Placement Using Intraoperative Computed Tomography-Based Navigation with Automated Referencing. Part II: Thoracolumbar Spine. Neurosurgery.

[21] Arun K.S., Huang T.S., Blostein S.D. (1987). Least-squares fitting of two 3-D point sets. IEEE Trans. Pattern Anal. Mach. Intell..

