# Supplementary material for: A Dual-Modal Hybrid Marker for Automatic Registration and Coordinate Hand-Over in C-Arm CT-Guided Spinal Navigation
Source: Sensors (Basel). 2026 Oct 12;26(20):6433. doi: 10.3390/s26206433 (PMC13709288; doi:10.3390/s26206433)
Supplement: Supplementary file 1 [file sensors-26-06433-s001.zip › Supplementary_Material_Sensors.pdf]

Supplementary Materials: A Dual-Modal Hybrid Marker for Automatic Registration and Coordinate Hand-Over in C-Arm CT-Guided Spinal Navigation

Taeho Kim and Sungmin Kim

S1. Operator-level TRE comparison

The analysis comprises 150 manual registrations (10 operators, three CT datasets and five trials per operator and CT) and one automatic benchmark per CT. Five fiducials determine the rigid registration. TRE is the RMS distance between the three held-out RF design centers, transformed using the saved registration matrices, and their corresponding CT centers.

Table S1. Individual operator contrasts. Each manual mean equally weights the three CT-specific five-trial means. The difference is the manual mean minus the fixed, equally weighted automatic mean (0.538 mm). All values are in millimetres. Values were rounded after calculation.

Operator	Manual mean TRE (mm)	Difference (mm)
P01	1.481	0.943
P02	1.495	0.958
P03	1.468	0.930
P04	1.459	0.921
P05	1.492	0.955
P06	1.479	0.941
P07	1.617	1.079
P08	1.570	1.033
P09	2.050	1.512
P10	1.538	1.001

The equally weighted means were 1.565 mm for manual registration and 0.538 mm for automatic registration. The mean manual-minus-automatic difference was 1.027 mm; all ten operator contrasts were positive. Table S1 shows the spread of individual operator differences, whereas the confidence intervals below describe uncertainty in their mean.

The 95% percentile bootstrap interval for the mean difference was 0.951-1.148 mm. The bootstrap used 10,000 resamples of the ten operators, retaining each operator's complete CT/trial bundle and keeping the automatic observations fixed. The t interval with nine degrees of freedom was 0.900-1.154 mm as a sensitivity analysis of the same mean. Both intervals are conditional on the three CT datasets and their observed automatic benchmarks.

S2. Fiducial-coordinate consistency

Manual fiducial selection was summarized using 250 coordinates per CT (10 operators, five trials and five fiducials). For each fiducial, pooled scatter is measured about its overall manual centroid. Within-operator scatter uses the operator-specific centroid; between-operator scatter describes those centroids about the overall centroid. Squared Euclidean distances are pooled across fiducials before taking the square root.

Table S2. Observed manual fiducial-coordinate summaries. All values are RMS distances in millimetres and use the original recorded coordinates. The final column compares manual coordinates with the observed automatic centers.

CT dataset	Pooled coordinate RMS (mm)	Within-operator RMS (mm)	Between-operator centroid RMS (mm)	RMS distance to automatic centers (mm)
C-arm CT	1.091	0.844	0.692	1.729
Conventional CT	1.065	0.828	0.669	1.817
PET/CT	1.120	0.829	0.754	1.709

Pooled coordinate scatter ranged from 1.065 to 1.120 mm. Within-operator scatter ranged from 0.828 to 0.844 mm, and between-operator centroid scatter from 0.669 to 0.754 mm, showing contributions from both repeated selections and differences between operators. Distances to automatic centers ranged from 1.709 to 1.817 mm. These automatic centers are comparison locations, not independently measured reference positions.

S3. Code and data reproduction

The supplementary materials include two files: this PDF and the code and data archive, *Supplementary_Code_and_Data_Sensors.zip*. The code archive contains the 153 trial records, fixed bootstrap indices, recorded detection traces, numerical evaluation code, and 10 full-precision reference CSV files. The displayed Tables S1 and S2 are generated from the full-precision results, with values rounded to three decimal places.

On Windows, extract the code archive and open RUN_REPRODUCE.bat. Its menu can prepare a package-local Python 3.12 environment with the pinned dependencies or reuse an existing one. The runner verifies input hashes, replays five-point FRE and three-target TRE using the actual saved transforms, and regenerates the statistical summaries, tables and figures. Successful execution writes generated/reproduction_summary.json with status PASS. Detailed data definitions and execution instructions are in the package README.

Reproduction starts from the saved coordinates and matrices; CT image acquisition, image detection and live tracking are outside this offline calculation. The recorded registration durations are analyzed as measured observations, rather than replaced by replay runtime. This preserves the manuscript's frame-center evaluation and timing definitions.
